# Endometriosis of the Inguinal Canal Mimicking a Hydrocele of the Canal of Nuck

**DOI:** 10.1155/2020/8849317

**Published:** 2020-09-08

**Authors:** Oshan Basnayake, Umesh Jayarajah, Sanjeewa Anuruddha Seneviratne

**Affiliations:** ^1^Professorial Surgical Unit, National Hospital of Sri Lanka, Colombo, Sri Lanka; ^2^Department of Surgery, Faculty of Medicine, University of Colombo, Sri Lanka

## Abstract

Isolated presentation of endometriosis of the inguinal canal is infrequent, and the clinical and imaging findings may be misleading in such patients. We describe an otherwise healthy female with isolated inguinal endometriosis presenting as a hydrocele of the canal of Nuck. Surgeons should consider such unusual presentations and obtain imaging and histological evaluations in doubtful instances. Complete excision was curative in our patient with no evidence of recurrence.

## 1. Introduction

Isolated presentation of endometriosis of the inguinal canal is infrequent. Clinical and imaging findings may be misleading in such patients. The presentation of inguinal endometriosis as a well-defined cystic inguinal swelling mimicking a hydrocele of the canal of Nuck is a rare occurrence. Therefore, we describe an otherwise healthy female with an isolated inguinal endometriosis with this unusual presentation.

## 2. Case Presentation

**Figure 1:**
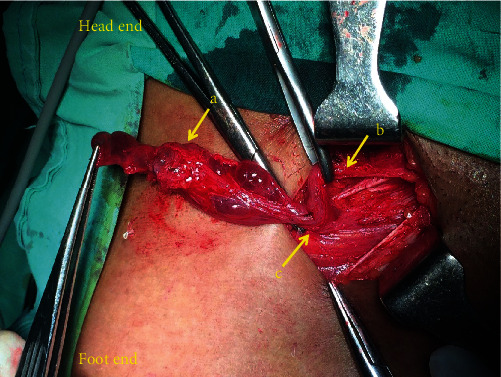
Intraoperative image of right inguinal canal. a—multiloculated cystic mass; b—round ligament of the uterus; c—deep inguinal ring.

A 27-year-old otherwise healthy Sri Lankan Sinhalese female presented to the surgical clinic in the year 2019 with an enlarging, painless, right inguinal swelling of 4 months duration. She was otherwise asymptomatic with no significant past medical or surgical history. She had regular menstrual cycles without any dysmenorrhea, dyspareunia, or history of infertility. She was not on any regular medication or combined oral contraceptive pills in the past. She did not have any past history of surgeries or gynecological procedures. Examination revealed an irreducible, 4 × 4 cm well-defined, cystic lump at the inguinal region without any skin changes. There was no tenderness. There was no cough impulse, and bowel sounds were absent. Clinically, a hydrocele of the canal of Nuck was suspected. Ultrasonography revealed a multiloculated, thin septated, anechoic cystic swelling without increased internal vascularity at the right inguinal region, and there was no demonstrable hernia, in favor of the clinical diagnosis. Her basic biochemical investigations were normal. After discussing with the patient, she underwent an elective inguinal exploration. There was a multiloculated cyst in the inguinal canal anterior to the round ligament of the uterus (Figure 1). A complete excision of the cyst was performed under spinal anesthesia, and the patient was discharged the following day (year 2019). The histology revealed endometrial glandular epithelium with hemosiderin-laden macrophages and background fibrosis suggestive of endometriosis. Immunohistochemical investigations were not performed in the specimen. She was referred to the gynecologists and was given a course of combined oral contraceptive pills. Follow-up after 1 year showed no evidence of recurrence, and the patient was asymptomatic.

## 3. Discussion

Endometriosis is defined as the presence of endometrial tissue in sites other than the uterine cavity. Although the majority of the patients have pelvic endometriosis, a considerable proportion also present with extra pelvic manifestations [1]. The involvement of anterior abdominal wall or inguinal region is usually related to previous surgery, secondary to implantation, and manifestation in these regions in the absence of previous surgery is a rare phenomenon [2].

The first case of inguinal endometriosis was reported in 1896 by Allen et al. [3]. Results from a systematic review evaluating extrapelvic endometriosis summarized 230 patients with anterior abdominal wall endometriosis [4]. Of this group, 58% had inguinal region involvement, and the majority presented with a lump in the inguinal region. All these patients had primary inguinal endometriosis similar to the patient reported here.

Since inguinal endometriosis mimics common surgical conditions of the region including inguinal hernia and hydrocele of the canal of Nuck, imaging modalities such as ultrasonography is helpful prior to surgery. These lesions can appear as hypoechoic masses with solid or cystic components [5]. The role of magnetic resonance imaging (MRI) in the diagnosis of pelvic and thoracic endometriosis has been studied and was found to have statistically significant accuracy compared with other modalities. However, the role of MRI in relation to abdominal wall endometriosis is uncertain [6]. A case of incidental finding of inguinal endometriosis in a whole body iodine-131 (I-131) uptake scan after total thyroidectomy has been reported [7]. Cancer antigen 125 (CA-125) levels are elevated in patients with endometrioses, but the role as a serum biomarker for the diagnosis lacks specificity [8].

Due to the lack of accurate diagnostic method and the mimicry of common inguinal conditions, most of the reported cases were managed initially by the surgeons [4]. The preoperative under diagnosis of inguinal endometriosis is commonly associated with atypical presentation as inguinal hernia or hydrocele of the canal of Nuck [9]. Some of the reported cases have shown inguinal hernia with concomitant inguinal endometriosis [10]. The final diagnosis is only confirmed by the histological presence of endometrial tissue in the excised lump or cyst. Mullerian epithelial tumor markers such as nuclear expression of PAX8 and interferon-inducible transmembrane protein-1 have been found to be highly sensitive in doubtful histological appearances [11, 12].

The risk of malignant transformation of endometrial deposit is between 0.7 and 1.0% [13] with only 6 cases of cancers associated with inguinal endometriosis have been reported in the literature [14, 15]. Of those, five out of six were adenocarcinomas, and one was an endometrial stromal sarcoma [16].

Of the extrapelvic endometriosis, the occurrence of inguinal endometriosis is rare. Misdiagnosis if common as the condition may mimic common inguinal conditions. Interestingly, the reported patient did not have any menstrual or cyclic symptoms. In doubtful cases, histopathological, evaluation would be helpful to clinch the diagnosis.

## 4. Conclusion

We describe an otherwise healthy female with isolated inguinal endometriosis presenting as a hydrocele of the canal of Nuck. Surgeon's should consider such unusual presentations and obtain imaging and histological evaluations in doubtful instances. Complete excision was curative in our patient with no evidence of recurrence.

## Data Availability

All data generated or analyzed during this study are included in this published article.

## Abbreviations

MRI: Magnetic resonance imaging
CA-125: Cancer antigen 125.
